# Evidence to Inform Policy and Practice: Mechanisms to Address Racial/Ethnic Disparities in Nursing Home Quality of Life

**DOI:** 10.1093/geroni/igac037

**Published:** 2022-05-23

**Authors:** Tetyana P Shippee, Heather Davila, Weiwen Ng, John R Bowblis, Odichinma Akosionu, Tricia Skarphol, Mai See Thao, Mark Woodhouse, Roland J Thorpe

**Affiliations:** Division of Health Policy and Management, School of Public Health, University of Minnesota, Minneapolis, Minnesota, USA; Center for Access and Delivery Research and Evaluation (CADRE), Iowa City VA Healthcare System, Iowa City, Iowa, USA; Division of General Internal Medicine, Carver College of Medicine, University of Iowa, Iowa City, Iowa, USA; Division of Health Policy and Management, School of Public Health, University of Minnesota, Minneapolis, Minnesota, USA; Department of Economics, Farmer School of Business, Miami University, Oxford, Ohio, USA; Division of Health Policy and Management, School of Public Health, University of Minnesota, Minneapolis, Minnesota, USA; Division of Health Policy and Management, School of Public Health, University of Minnesota, Minneapolis, Minnesota, USA; Department of Religious Studies and Anthropology, University of Wisconsin–Oshkosh, Oskhosh, Wisconsin, USA; Division of Health Policy and Management, School of Public Health, University of Minnesota, Minneapolis, Minnesota, USA; Hopkins Center for Health Disparities Solutions, Johns Hopkins Bloomberg School of Public Health, Baltimore, Maryland, USA

**Keywords:** Case study, Equity, Long-term care, Mixed methods, Person-centered care

## Abstract

**Background and Objectives:**

Abundant evidence documents racial/ethnic disparities in access, quality of care, and quality of life (QoL) among nursing home (NH) residents who are Black, Indigenous, and people of color (BIPOC) compared with White residents. BIPOC residents are more likely to be admitted to lower quality NHs and to experience worse outcomes. Yet, little is known about processes for differences in QoL among residents receiving care in high-proportion BIPOC NHs. This study presents an examination of the processes for racial/ethnic disparities in QoL in high-proportion BIPOC facilities while highlighting variability in QoL between these facilities.

**Research Design and Methods:**

Guided by the Minority Health and Health Disparities Research Framework and the Zubritsky framework for QoL in NHs, we employ a concurrent mixed-methods approach involving in-depth case studies of 6 high-proportion BIPOC NHs in Minnesota (96 resident interviews; 61 staff interviews; 614 hours of observation), coupled with statewide survey data on residents’ QoL linked to resident clinical Minimum Data Set assessments.

**Results:**

Quantitative findings show that BIPOC residents experience lower QoL than White residents across various domains. Qualitative findings reveal variability in BIPOC residents’ QoL between high-proportion BIPOC facilities. In some facilities, BIPOC residents experienced worse QoL based on their race/ethnicity, whereas in others BIPOC residents QoL was not directly affected by their race/ethnicity or they had mixed experiences.

**Discussion and Implications:**

The findings highlight variability in racial/ethnic disparities in QoL across NHs with a high proportion of BIPOC residents. We identify health equity initiatives, including engaging with community BIPOC organizations and volunteers, and providing more resources to high-proportion BIPOC facilities to support staff training, additional staffing, and culturally specific programming. Given the increasing racial/ethnic diversity of NHs, ensuring equity in QoL for BIPOC residents is an urgent priority for NHs to remain relevant in the future.

**Translational significance:** Nursing homes need to address racial/ethnic disparities in quality of life to meet the needs of a growing proportion of residents who are Black, Indigenous, and persons of color (BIPOC). This is the first study that examines processes for differences in QoL among residents in NHs with a high proportion of BIPOC residents and identifies initiatives that could be undertaken to make long-term care delivery more equitable.

Long-term care (LTC) is the most racially segregated form of health care in the United States (1). Furthermore, abundant evidence documents racial/ethnic disparities in access, quality of care, and quality of life among nursing home (NH) residents who are Black, Indigenous, and people of color (BIPOC) compared with White residents (1–3). BIPOC residents are more likely than White residents to be admitted to lower quality NHs and experience adverse events and lower quality of life (QoL) (4). The United States now has the most racially/ethnically diverse NH population in the country’s history (5), creating a long-overdue impetus for change in how LTC is delivered to BIPOC residents.

Several studies have attributed disparities experienced by BIPOC NH residents to being admitted to poorer-quality facilities versus within-facility variability (1,4). Recent studies point to systemic racism as a root cause of these disparities, manifested via racial segregation of care and other pathways (6). Systemic racism is broadly operationalized as a system of “structuring opportunity and assigning value based on race/ethnicity” that unfairly disadvantages some individuals and communities and unfairly advantages others (7). Systemic racism in LTC has operated through segregation and BIPOC residents more often being admitted to higher proportion BIPOC resident facilities (8) that have attributes associated with worse quality, such as being primarily Medicaid-reimbursed or for-profit ownership. For example, Chang et al. found that 90% of Black residents in Missouri were concentrated in 20% of the state’s NHs. Furthermore, the more racially segregated NHs were, the poorer the quality of care (eg, pressure ulcers, vaccinations) was for residents (9).

Although most studies have focused on quality of care (1,4,9,10), more recent studies have also started to examine QoL. QoL is a person-centered measure of overall well-being that includes person–environment fit, attention from staff, meaningful relationships and activities, and enjoyment of food (11). A growing body of work recognizes QoL as another fundamental aspect of NH quality. There is no national measure of QoL, but Minnesota and Ohio administer validated QoL surveys annually (11). A study using Minnesota’s QoL measure found, controlling for health status and facility characteristics, BIPOC residents had lower QoL scores than White residents in most QoL domains, as did all residents of high-proportion of BIPOC facilities (12). Moreover, facility characteristics and receiving care in high-proportion BIPOC NHs explained a much greater share of the BIPOC-White disparity than individual characteristics (13).

Despite these important quantitative results, we know very little about mechanisms through which systemic racism operates in creating disparities in NH QoL. Although BIPOC residents are more likely to be admitted to high-proportion BIPOC NHs, which are different than other NHs in numerous ways (eg, more often for-profit, high-proportion Medicaid payments (13)), no study has examined processes of care or between-facility differences among high-proportion BIPOC resident NHs. This study presents a comprehensive mixed-methods examination of the processes for racial/ethnic disparities in QoL in high-proportion BIPOC facilities.

## Theoretical Framework

We were guided by the National Institute on Minority Health and Health Disparities (NIMHD) Health Disparities Research Framework (14) and Zubritsky et al.’s framework for QoL for LTC (15). The NIMHD framework (14) helps identify levels of influence whereby systemic racism affects NH residents’ QoL (and areas of intervention): *individual*, *interpersonal/organizational*, *community*, and *societal*. For this article, we focus on systemic racism’s impacts on QoL via 3 levels—individual, interpersonal, and community—in the domains of *physical/built and sociocultural environments as they pertain to the NH environment.* The *individual level of influence* includes relationships between residents; the *interpersonal/organizational level* includes resident care and treatment by facility staff, social activities, and meals; the *community level* includes facility interactions with the broader community including community organizations and the role of community factors for staff retention. Zubritsky’s model for QoL considers how individual and facility factors influence each other, including race/ethnicity, language, and particular facility attributes, such as payment type, size, and location (15). Yet, Zubritsky’s et al.’s framework does not focus on care needs of those with marginalized lived experiences. To address this, we draw on both models to examine how individual, organizational, and community levels of influence shape QoL for BIPOC residents in high-proportion BIPOC NHs.

## New Contribution

Although prior studies found racial/ethnic disparities in NH residents’ QoL, including in the state examined in this study—most have been quantitative, other than a few single case studies on specific QoL domains. Prior studies have focused on individual resident responses and experiences without explicitly considering facility context as a key factor. We build on this work by carrying out case studies of 6 high-proportion BIPOC NHs in Minnesota to examine processes for BIPOC residents’ QoL. Specifically, our goals were to (a) identify processes resulting in racial/ethnic disparities in QoL in high-proportion BIPOC facilities and (b) examine *how* individual, organizational and community factors affect NH residents’ QoL and the role between-facility variation plays in these disparities.

## Method

### Study Overview

This is a multicase study analysis involving 6 high-proportion BIPOC NHs to examine processes for racial/ethnic disparities in NH residents’ QoL. We used a concurrent mixed-methods design, with quantitative data providing context and description for the qualitative data (quan-QUAL) (16). First, we started with quantitative data to identify high-proportion BIPOC facilities in Minnesota. We then used qualitative methods to recruit and carry out case studies of a purposive sample of 6 such facilities. Second, we used quantitative methods to analyze QoL from a statewide survey for BIPOC versus White residents in these facilities (compared with a statewide sample), whereas we simultaneously used qualitative methods to elucidate levels of influence on QoL for BIPOC residents in these facilities. We then interpreted quantitative and qualitative results together in our comparison of the 6 facility cases. The study was approved by the University of Minnesota Institutional Review Board.

#### Definition of high-proportion BIPOC facility

We used quantitative data from the Minimum Data Set (MDS) from 2017 to identify facilities for the qualitative sample based on proportion of BIPOC residents. BIPOC grouping includes those who are Black and American Indian and “other” from communities of color (which combined Asian, Hispanic/Latinx, and multirace due to smaller sample sizes). We defined *high-proportion BIPOC facilities* as those in at least the 90th percentile by proportion of BIPOC residents in Minnesota, which was 17.7% in 2015. Based on these criteria, we used purposive sampling to select the 6 facilities that were in the 90th percentile, ensuring that we had facilities with different ownership (for-profit/not-for-profit even split), ethnic/race mix (eg, primarily Black vs primarily Asian), and variability in the proportion of BIPOC residents to allow comparisons. The BIPOC proportions in our sample range from 17.6% to 53.8%. Although BIPOC is a broad term, we focused on the importance of concentration of historically marginalized racial/ethnic groups at the facility level, particularly in a state that is predominantly White and known for progressive policies alongside high health disparities. In qualitative analyses, we refer to each respondent’s specific self-identified racial/ethnic group.

### Qualitative Methods

#### Facility recruitment

Facility recruitment was facilitated through partnership with provider association representatives in Minnesota. All of the selected 6 facilities agreed to participate (100% response rate). We gave facilities a $1 500 incentive to participate. Once a facility agreed to participate, the research team met with facility leadership, including social workers, to discuss enrollment criteria and methods of recruitment.

#### Data collection

Between November 2016 and April 2019, we conducted data collection in the 6 facilities. We conducted semistructured interviews with 96 long-stay residents and 61 staff, and 618 hours of observation designed to be complementary. Interviews were conducted by members of the research team who conducted observations and hence were familiar with residents/staff. We obtained informed consent for all interviewees.

##### Interviews with residents

We purposively sampled residents to achieve diversity in race/ethnicity and gender, among other factors. Based on the resident census, the research team distributed recruitment flyers to eligible residents, then followed up with a request for an interview; we had a 94% resident response rate (*n* = 96). Residents in the NH less than 30 days or who had severe cognitive impairment were excluded. Most interviews were in English, but 11 were conducted in Hmong or Vietnamese, then translated into English using established best practices (17). Resident participants included similar numbers of men and women (45 women and 51 men) and reflected the racial/ethnic diversity of facilities (39 African American, 27 White, 8 Hmong, 4 Vietnamese, 7 Native American/American Indian, 8 African, 2 Hispanic/Latino, and 1 multirace). The average interview length was 34 minutes (range: 14–73) and focused on a variety of topics concerning QoL (see resident interview guide in Supplementary Material).

##### Interviews with staff

Staff were purposively sampled to include different roles (administrators, Directors of Nursing, Certified Nurse Assistants, etc.) and achieve a racially diverse pool. We met with key facility staff (eg, social services, nursing, activities) to explain the study; the facility administrator also sent an email to staff describing study procedures. Eligible staff were invited via recruitment flyer, with research staff following up with a request for an interview; 90% (*n* = 61) agreed to participate. Of the 61 staff members interviewed, 57% (*n* = 37) were BIPOC including 13 African American, 10 of African descent, 6 Hmong, 1 Native American, 4 Vietnamese or other Asian descent, and 3 other multiracial. Average interview length was 38 minutes (range: 15–66 minutes). Interview questions focused on relationships between staff and residents and staff perception of whether race/ethnicity impacted residents’ QoL. A copy of the interview guide is in Supplementary Material.

##### Observations

The observations provided important context for understanding residents’ lived experiences, establishing rapport between research team, residents, and staff, and facilitating triangulation of findings (18). Observations spanned 4 months in each facility. Members of the research team visited the facility twice weekly during varied hours, spending on average 4 hours per visit. Observations were primarily unstructured and focused on understanding residents’ experiences that could influence their QoL. Team members talked with residents, helped with activities, and observed activities and meals. Daily, each observer typed detailed notes based on their observations and reflections.

#### Qualitative analysis

Interviews were professionally transcribed. We used a partially deductive/partially inductive approach to coding interviews and observation notes. We used thematic analysis for case studies (19). The initial coding framework was based on QoL domains included in the quantitative QoL measure and the role of race/ethnicity on residents’ QoL. We also used an inductive approach to identify additional themes found that did not fit existing domains. Two investigators independently coded all data. To enhance the validity of findings, a third investigator who was familiar with the NHs and participated in data collection served as an external reviewer for inductively generated themes (20). Once coding was finalized, we generated case summaries of each facility via a team process for consensus-building and to allow comparison across sites (19). We used NVivo qualitative software for data management.

#### Defining 3 facility groups

Using qualitative findings, we classified facilities as a team into groups based on the role that race/ethnicity played in residents’ QoL within facilities. We developed these groups based on detailed case summaries of each facility, drawn from extensive observations and staff/resident interviews. Triangulating across qualitative sources, qualitative results guided our classification of facilities into 3 groups. Facilities where race/ethnicity greatly influenced BIPOC residents’ QoL were classified as “*high-disparity*” (3 facilities); facilities where race/ethnicity did not greatly influence BIPOC residents’ QoL as “*low-disparity*” (2 facilities); and one where BIPOC residents had mixed experiences with QoL based on race/ethnicity as “*mixed results*.”

### Quantitative Methods

#### Data

We used 3 sources of data: resident QoL surveys, linked with MDS data to obtain respondent race/ethnicity and other aggregated resident characteristics, and Minnesota Medicaid Cost Reports for facility-level characteristics. All quantitative data are from 2017 to coincide with qualitative data collection.

##### Resident QoL surveys

We used a validated QoL instrument collected in a random sample of residents in Medicaid-certified NHs in MN by a survey firm. An average of 35 long-stay (defined as over 30 days) residents per facility were selected to participate. The instrument contains 48 items, representing 8 domains: activities, food enjoyment, environment, dignity, autonomy, relationships, caregiving, and mood (11,21). We calculated standardized sum scores of individual items for each domain (0–100; higher values indicate better QoL). We also calculated summary scores, averaged for the 8 domains.

Supplementary Table 1 provides a brief description of each domain. Residents were excluded if they had severe cognitive impairment or significant behavioral symptoms, were in medical isolation, guardian-declined participation, or communication barriers prevented participation (22).

##### Resident race/ethnicity

Residents who reported Hispanic ethnicity or identified as Black, Indigenous, or from other communities of color on the MDS were grouped under BIPOC category. Despite important heterogeneity within this group, we were limited in subgroup analyses due to small sample sizes. We reported the *proportion of BIPOC residents* for each facility group, as well as the proportion of residents who identified as Asian, Black, Hispanic, and American Indian race/ethnicity.

##### Resident and facility characteristics for each facility group

We identified resident and facility characteristics in each group (“high disparity,” “low disparity,” “mixed results” versus all facilities in the metropolitan area) based on the Zubritsky et al.’s framework (15) and other literature as impacting QoL (21).

Resident characteristics were aggregated facility level and include the proportion of residents who were female, were under age 65, had a diagnosis of Alzheimer’s disease or related dementia, and had a mental health diagnosis other than depression (including schizophrenia or related conditions and bipolar disorder). Last, we calculated average physical function for residents in our sample facilities, measured on a 0–28 Activities of Daily Living scale (higher = greater impairment).

Facility characteristics included ownership (for-profit/not-for-profit and government); chain affiliation; proportion Medicaid, Medicare, and other payer resident days; number of beds; proportion of private rooms; and occupancy rate. We calculated staff hours per resident day for registered nurses, licensed practical nurses, certified nursing assistants, activities staff, and social workers separately and combined, and measured staff retention (percent of staff not leaving the facility each year) for all direct care staff (nurses and aides), nurses alone, and all staff combined. We also reported the number of total deficiencies and deficiencies based on complaints the facilities incurred.

#### Quantitative analysis

First, we compared facility and resident characteristics across the 3 facility groupings and compared them to all facilities in the metropolitan area. We used the metropolitan area for comparison because all facilities in the qualitative sample were from the metropolitan area. Next, we computed mean QoL for BIPOC versus White residents and compared across the 3 facility groups identified in qualitative analyses. We also calculated standardized mean QoL scores for BIPOC versus White residents for the statewide sample as a comparison. We carried out sensitivity analyses by residents’ individual race/ethnicity classified as Black non-Hispanic (majority of the BIPOC sample), all other BIPOC racial/ethnic groups, and White in determining QoL scores, shown in Supplementary Table 2. Results were very similar. We used Stata 16 to carry out quantitative analyses.

## Results

Table 1 shows facility and resident characteristics for the 3 facility groups (“high disparity,” “low disparity,” and “mixed results”), facilities of all 3 groups together, and metropolitan facilities overall. Facilities in the study population (the 6 facilities across the 3 facility groups) were different from metropolitan facilities overall: all were chain facilities, had much higher Medicaid payer-mix, lower direct care and activities staffing, more deficiencies, and residents more likely to be BIPOC group, male, under 65, with mental health diagnoses, and better functional health. Most BIPOC residents in the 6 high-proportion BIPOC facilities were Black, followed by Asian. Differences in resident characteristics compared with metropolitan facilities overall were most pronounced in the “high-disparity” group. “High-disparity” group facilities were most likely to be high-proportion Medicaid and were all for-profit; their residents were more likely to be male, younger, and have mental health diagnoses.

**Table 1.** Facility Characteristics by Group

Facility Groups*	High Disparity (*n* = 2)	Low Disparity (*n* = 3)	Mixed Results (*n* = 1)	All Interview Facilities (*n* = 6)	Ref. Group: All Metro Facilities (*n* = 122)
Resident characteristics (census)					
% BIPOC	41%	30%	23%	34% (range 23%–54%)	11% (range 0%–54%)
% Asian	10%	1%	1%	5% (range 0%–29%)	1% (range 0–29%)
% Black	22%	25%	14%	22% (range 14%–32%)	7% (range 0%–35%)
% Hispanic	5%	1%	1%	3% (range 1%–8%)	1% (range 0%–8%)
% American Indian	3%	2%	7%	3% (range 1%–8%)	1% (range 0%–14%)
% Female	35%	58%	55%	46% (range 25%–59%)	63% (range 0%–100%)
Mean age	64.9	76.3	73.8	70.1 (range 62–77)	78.0 (range 53–90)
% Under age 65	48%	14%	20%	32% (range 13%–55%)	18% (range 0%–86%)
% With ADRD diagnosis	26%	45%	40%	35% (range 15%–57%)	42% (range 0%–74%)
% With mental health dx other than depression	50%	39%	47%	46% (range 30%–65%)	37% (range 7%–99%)
Mean ADL28 score (higher = greater impairment)	9.6	12.6	12.6	11.1 (range 8.1–13.6)	14.7 (range 0.2–19.9)
Facility characteristics					
For-profit owner	100%	0%	0%	50%	44%
Part of chain	100%	100%	100%	100%	74%
Medicaid payer share (ResidentDays)	84%	76%	65%	78% (range 65%–89%)	55% (range 0%–100%)
Medicare payer share (ResidentDays)	6%	6%	11%	7% (range 3–11%)	13% (range 0%–74%)
Other payer share (ResidentDays)	2%	14%	7%	5% (range 1%–10%)	19% (range 0–68%)
Size (LicensedBeds)	98.7	188.5	190.0	143.8 (range 69–250)	104.7 (range 17–320)
Share of beds in private rooms	6%	22%	8%	11% (range 5%–26%)	38% (range 0%–100%)
Occupancy (Resident Days/CapacityDays)	88%	92%	87%	89% (range 85%–92%)	86% (range 35%–99%)
Staffing: hours per resident day, all direct care staff	3.69	4.26	4.10	3.95 (range 3.2–4.3)	4.56 (range 1.7–10.9)
Staffing: hours per resident day, registered nurse	0.55	0.76	0.73	0.65 (range 0.3–0.9)	0.60 (range0.1–5.8)
Staffing: hours per resident day, licensed practical nurse	0.73	0.64	0.48	0.66 (range 0.4–1.0)	0.68 (range 0–1.6)
Staffing: hours per resident day, certified nurse aides	1.81	2.06	2.30	1.97 (range 1.7–2.3)	2.50 (range 0.0–4.4)
Staffing: hours per resident day, social work	0.17	0.16	0.13	0.16 (range 0.1–0.2)	0.13 (range 0.0–0.5)
Staffing: hours per resident day, activities	0.14	0.25	0.14	0.18 (range 0.1–0.4)	0.27 (range 0.0–0.5)
Staffing: retention, all direct care	58%	73%	82%	67% (range 43%–82%)	67% (range 39%–91%)
Staffing: retention, all nursing	45%	79%	87%	63% (range 11%–87%)	67% (range 11%–100%)
Staffing: retention, aides	69%	67%	80%	70% (range 59%–80%)	66% (range 32%–100%)
Health and safety: number of deficiencies (NH Compare Cycle1)	9.3	12.0	8.0	10.0 (range 3–16)	7.3 (range 0–38)
Health and safety: number of deficiencies from Complaints (NHC Cycle1)	1.0	0.0	0.0	0.5 (range 0–2)	0.6 (range 0–9)

*Notes:* ADRD *=* Alzheimer’s disease and related dementias; BIPOC = Black, Indigenous, and people of color.

*Facilities where BIPOC residents reported that their race/ethnicity greatly influenced their QoL were classified as “*high disparity*” facilities; facilities where BIPOC residents reported that race/ethnicity did not greatly influence their QoL as “*low disparity*” facilities; and facilities where BIPOC residents reported mixed experiences about how race/ethnicity influenced their QoL as “*mixed results*.”

Using *t*-tests, we compared summary QoL for BIPOC versus White residents across the 3 facility groups and for the statewide sample as a comparison (Table 2). BIPOC residents reported lower overall QoL than White residents: a difference of 4.84 points (70.18 vs 75.02) in the “high-disparity” group compared with 1.63 (68.19 vs 69.82) in the “low-disparity” group. In the “mixed-results” facility, the overall QoL score was barely higher among BIPOC residents (0.82 points) than White residents. In the statewide sample, BIPOC residents reported 6 percentage points lower scores than White residents (73.49 vs 79.83, *p* < .001). *Food enjoyment* was the domain with the greatest disparity: 22 percentage-point difference for BIPOC versus White residents in the “high-disparity” group (*p* < .05) versus 12 percentage points in the “low disparity” group. This was also true for the full state sample (−11 percentage points, *p* < .001). *Caregiving from staff* and *activities* also had considerable score differences. These patterns all held for the statewide sample. Yet, differences for BIPOC versus White residents within each of these groups were only statistically significant for food enjoyment, likely due to small sample size. Also, BIPOC residents in the “mixed-results” facility had lower negative mood and higher dignity compared with White residents (not significant). In the statewide analyses, we found significant racial differences for all QoL domains, with BIPOC residents having lower scores (*p* < .001).

**Table 2.** Nursing Home Residents’ Quality of Life Scores by Race/Ethnicity for 3 Facility Groups, 2017 (Compared With All Nursing Homes in MN)

Variable	High-Disparity Group			Low-Disparity Group			Mixed-Results Group			State-Level Analysis		
	BIPOC Residents (*n* = 37)	White Residents (*n* = 62)	Diff.	BIPOC Residents (*n* = 31)	White Residents (*n* = 57)	Diff.	BIPOC Residents (*n* = 11)	White Residents (*n* = 34)	Diff.	BIPOC Residents (*n* =604)	White Residents (*n* = 9,225)	Diff.^†^
Overall QoL^†^	70.18	75.02	−4.84	68.19	69.82	−1.63	73.00	72.18	0.82	73.48	80	−6***
Food	49.76	71.76	−22.00**	52.33	64.29	−11.97	63.88	64.14	−0.27	66.56	78	−11***
Environment	83.49	83.28	0.22	83.16	81.07	2.09	85.43	87.18	−1.75	84	88	−4***
Dignity	85.75	86.67	−0.92	83.15	85.96	−2.81	91.07	83.45	7.61	88	94	−6***
Autonomy	76.78	75.83	0.95	76.68	73.66	3.02	79.43	73.44	5.98	78	83	−5***
Relationships	67.76	71.03	−3.26	63.28	62.76	0.52	60.60	66.68	−6.08	69	73	−4***
Caregiving	65.65	72.01	−6.36	65.67	66.01	−0.34	69.95	67.30	2.65	73	81	−8***
Activities	69.12	74.68	−5.56	63.31	62.34	0.97	64.21	72.01	−7.80	70	77	−7***
Lack of negative mood	62.60	59.72	2.49	53.89	59.92	−6.03	65.33	54.33	11.01	63	68	−5***
Positive mood	64.93	75.17	−10.24	66.05	67.66	−1.60	77.74	81.10	−3.36			

*Notes*: BIPOC = Black, Indigenous and people of color; QoL = quality of life. We present standardized means for sum scores of individual items for each of the 8 domains of QoL. The scores range from 0 to 100; higher values indicate better QoL. We also calculated overall summary score; the average standardized scores for the 8 domains.

^†^All analyses for state-level comparisons are significant at *p* < .001.

### Qualitative Themes on Resident QoL

Based on the Minority Health and Health Disparities Framework (14) and the Zubritsky et al.’s framework (15), we identified levels of influence driving racial/ethnic disparities in BIPOC residents’ QoL and how they differed between high-proportion BIPOC facilities. Table 3 displays results by the 3 facility groups.

**Table 3.** Qualitative Results Summarized by Levels of Influence by Facility Group

Groups	Levels of Influence per NIMHD Framework		
	Individual (Theme 1)	Organizational (Theme 2)	Community (Theme 3)
High-disparity group	Resident relationships: Tensions among BIPOC and White residents; segregation based on residents’ race/ethnicity; frequent use of racial slurs; instances of aggression.	Care from staff: BIPOC residents described receiving inferior care vs White residents or residents who had racial/ethnic or cultural similarity with staff. Activities: Few options for engagement; most common activity was bingo. BIPOC residents said activities were not culturally sensitive. Food: Most BIPOC residents were highly dissatisfied with food, which they described as bland and White-centric.	Engagement with BIPOC communities and volunteers: Most facilities had no volunteers with very few exceptions. Lack of engagement with BIPOC community groups (despite being located in racially/ethnically diverse neighborhoods), led to lack of transparent culture of care and fewer options for residents’ engagement.
Low-disparity group	Resident relationships: Many BIPOC and White residents got along. Some noted issues with immigrant residents with limited English proficiency, especially related to communication.	Care from staff: Most residents describe care as “equal” across race/ethnicity. Diversity is viewed positively. Activities: Facilities invested in a variety of activities, including by providing transportation to take residents on outings and having dedicated indoor and outdoor spaces for activities and gardens. Food: Although residents had concerns about food and mistakes were made, residents said facility leadership was working to make changes.	Engagement with BIPOC communities and volunteers: Strong volunteer presence and infrastructure to support ongoing community engagement.
Mixed-results group	Resident relationships: BIPOC residents had mixed reactions if race/ethnicity was a factor in relationships. Most White residents said it was not a factor.	Care from staff: Many residents said race/ethnicity did not affect care, although some differences based on racial/ethnic concordance noted. Activities: Residents said more activities were needed, especially on weekends but many residents were satisfied with activities offered. Food: Mixed experiences with food, but efforts were made to improve offerings.	Engagement with BIPOC communities and volunteers: Limited community involvement, with a few volunteers and primarily church groups on Sunday.

*Notes:* BIPOC = Black, Indigenous, and people of color; NIMHD = National Institute on Minority Health and Health Disparities.

*Per NIMHD framework, societal level of influence is also important but is outside the scope of our focus for this article.

**p < 0.01. ***p < 0.001.

#### Individual level of influence: relationships between residents

##### High-disparity group

When asked if race/ethnicity affected residents’ relationships, many residents in this group reported residents were self-segregated based on race/ethnicity and language. A resident said:

No, I talk to Vietnamese residents only. I don’t know the language to communicate with people of other race/ethnicities. Vietnamese resident (Facility 1)

Observations supported this sentiment: residents from different racial/ethnic groups often did not engage with each other, despite living on the same floor. Residents in these facilities described cliques, many based on race/ethnicity, pointing to self-segregation among residents.

As one African resident commented:

Somebody didn’t like me because I was a foreigner. African resident (Facility 5)

Observations supported these findings, with notes describing immigrant residents and many BIPOC residents often sitting alone in their room for long periods, alone at dinner, and often not joining any activities. For example, we observed an African-born resident sitting in the lobby most days; we often saw him observing others coming to the facility, walking around the facility and not having activities or friends with whom he engaged. Similarly, a Native American resident told our team member that she was very lonely: she had no family visiting her and said she felt she had no friends in the facility. She had several people with whom she sat for meals but rarely talked with after that. We observed this same dynamic for non-U.S.-born residents, most of whom were BIPOC residents, with staff not engaging with non-English-speaking residents or encouraging them to join activities. We also observed instances of aggressive behavior among residents and instances of racial slurs used by White residents in the presence of BIPOC residents. For example, one resident commented:

I think there was one guy who wished to disturb here. He used to go all like, he insulted people, [used] racial slurs. African resident (Facility 5).

Numerous residents also described serious issues (eg, illicit drugs) creating an unsafe environment. Site observations similarly revealed issues regarding substance use and at times unsanitary conditions (eg, mice infestation). Many residents told us that boredom was the biggest reason for their illicit drug use, with particular salience for younger male residents. As some staff told us, to avoid disruption and aggression among some residents, policies were not enforced unless the use was egregious. In at least one instance, staff discussed eviction of one resident using illegal substances due to the trouble they were causing. In another, a resident smuggled in alcohol and became intoxicated with another resident, who then refused to take any medication, and acted belligerently toward staff.

##### Low-disparity group

Residents in these facilities overwhelmingly said race/ethnicity was not a factor in resident relationships, with comments like “everyone mixes” or “everyone gets along.” These sentiments were similar across BIPOC racial/ethnic groups. When probed about whether they noticed any tensions between residents due to race/ethnicity, one resident said:

No, no, we don’t talk about no race/ethnicity. They don’t talk about no race/ethnicity around this place. Everybody get along like sisters and brothers around here. African American resident (Facility 2)

Although most residents in this group said race/ethnicity was not a factor in residents’ relationships, several residents described some divisions associated with language barriers or immigration status, as the following comment illustrates:

But the Somalis, I think the problem is not, there is no hatred, but the language. African resident (Facility 4)

From observations, research staff observed that Somali residents did not attend facility-run activities, instead typically gathering on their own. In some instances, this included Somali men having regular dominos sessions. We also observed a group of female White residents who always sat together for meals and would not allow others to join their table. Some activities were primarily attended by White residents, whether due to selection, self-withdrawal of those from other racial/ethnic groups, or intentional “boundary” maintenance by the social group.

##### Mixed-results facility

Although residents in this group had some positive comments about relationships with other residents, some residents made neutral comments, such as “some do [get along], some do not” or “I think they do for the most part, [residents] get along quite well.” Some BIPOC residents in this group said that residents of the same race/ethnicity tended to get along better. A larger number of White residents said race/ethnicity was not a factor, whereas BIPOC residents expressed more varied views. From observations, some BIPOC residents felt more comfortable discussing informally with researchers that they felt administration and some residents were racist or “had bad apples” using racist language but did not want to go on record with a formal recorded interview saying so.

#### Organizational level of influence: care from staff, opportunities for meaningful activities, and food-related issues

##### Care from staff

###### High-disparity group

Residents and staff in this group described racial concordance between residents and staff as an important factor in receiving good care, although language concordance was also important. Some residents said staff took care of English-speaking residents from their own racial/ethnic group first, such as one resident who commented:

They [staff] try to treat good. But to say it, the people [who can’t speak English] wait for the last minute and they take their people first. Hmong resident (Facility 1)

Another resident described being treated differently by staff based on her race/ethnicity (there were no direct care Vietnamese staff members at that NH):

They can shower everyone. But when it’s my turn, sometimes, there is no one showering me. It’s difficult if I have to request a shower. I am showered two times a week. Vietnamese (Facility 1)

Several BIPOC staff in these facilities described being subjected to racial slurs by residents (similar to those experienced by BIPOC residents in Theme 1). Both residents and staff described some language barriers with foreign-born staff (such as several residents who said they had difficulty understanding and communicating with African staff).

Despite caring for many BIPOC and immigrant residents with limited English proficiency, staff said they received no training and little to no support from facility leadership in providing culturally sensitive care, as described by one staff member:

Training is always good, and why not? Since when we are talking about racial disparity, yes, how do we...do we provide care when it comes to, are there any programs that are geared towards caring for residents that are racially different than say, Caucasian? African staff (Facility 5)

Several residents said facility administration applied different standards to BIPOC residents and White residents, such as BIPOC but not White residents who engaged in altercations or illicit drugs facing consequences. When asked if administration listens to them, an African American resident responded: “Well, some…they just help their own. Me? No, they do not.”

We observed BIPOC residents talking about needing to advocate for themselves and other BIPOC residents to get help resolving issues in care. Some BIPOC staff felt that administration was inept and that staff had to advocate for BIPOC residents. In one instance, a BIPOC resident told us and a staff member that they reported a complaint to administration about residents’ care concerns but nothing was done. These issues contributed to a lack of trust between residents and administration.

###### Low-disparity group

In this group, residents and staff across racial/ethnic groups generally said race/ethnicity did not influence how care was delivered or, in the words of one resident, that it was “equal” (African American resident, Facility 2). Several respondents said they appreciated racial/ethnic diversity, even if there were some language-related challenges. According to one resident:

I just love that [facility] has all these new people from Africa and they’re from all over. And that’s one of the problems too. All the different languages. Not just Somali, it’s Kenyan and it’s Ethiopian…a lot of Ethiopians. And I love almost every one of them that I’ve met here, the workers. White resident (Facility 4)

A staff member similarly commented:

Our environment, I think it’s good. We have a mesh of young people, old people, people from different backgrounds, everywhere. But I think everyone meshes together really well…I don’t know what we’d do without each other up here. African staff member (Facility 2)

Another staff member alluded to the importance of an organizational culture that values diversity:

One of the biggest things I appreciate here that they...[facility] is the first place I’ve ever worked at that takes culture, everybody’s culture, into perspective, and respects it. African American staff member (Facility 4)

We observed instances where BIPOC staff in management positions in this facility took more time to listen to and value opinions of BIPOC residents, including when they were in a hurry to another meeting or had other demands.

###### Mixed-results facility

Most residents in this group said race/ethnicity was not a key issue in staff care. Yet, there were some exceptions, with some residents saying residents received better care when they shared the same racial/ethnic background as staff. One resident commented:

My nurse, she’s Native, and I get the care. My light goes on and she’s doing…she’ll come and see what I want. But with my aide, she’s Black, she don’t do nothing…Native American resident (Facility 3)

Another resident similarly said:

I’ve seen some of the Black aides will pay a little more attention to Black residents. White resident (Facility 3)

Although most residents indicated race/ethnicity did not affect care, several respondents (particularly staff) said language barriers made caregiving more challenging, such as a staff member who said:

I know one issue is language barriers. We’ve had some Somali speaking patients and Spanish speaking. That makes it difficult because you can’t really understand what they’re trying to say. Then at times, we do get interpreters, so that definitely does help. White staff member (Facility 3)

##### Opportunities for meaningful activities

###### High-disparity group

Interviews and observations revealed residents in these facilities had few options for activities other than bingo. When residents were asked what activities they would like, the overwhelming response was to have more options offered, particularly community outings (eg, to movies, museums, parks). Residents described the challenge of meeting the needs of residents of different ages, abilities, and racial/ethnic backgrounds. One resident commented:

There’s nothing to do, play cards, back, play cards, back. I wanted them to try to juice up the schedule, get a focus group together so we could have to choose. Because you got younger people here that don’t want to just sit and play bingo with half of the folks who don’t even know what’s going on. African American resident (Facility 6)

A staff member said:

I just don’t think they’ve got a lot of Black things, you know, that they would really like. They’ve got a lot of White games. African American staff (Facility 5)

###### Low-disparity group

Residents in these facilities had more activity options, including community outings, and more physical space for activities (eg, large activities room). Numerous residents described the importance of facilities providing transportation to parks, shopping, and other places. These facilities also had outside gardens and other activities (eg, singing). One resident commented:

I am a member of the garden club. I’m very fascinated by the different plants and planting different plants. That’s very important to me. African American resident (Facility 2)

A resident in another facility said:

I go out with the group at least once a month. And sometimes I go to the trivia…And sometimes I go to the singalong…And they have a library up on [floor number] and I get in my wheelchair and go up there…Well they have those what they call the spring breakfast and some family comes and... African American resident (Facility 4)

Having space and services available to support residents’ religious and spiritual needs was another important finding in low-disparity facilities. One facility had created a prayer room, which residents and staff said they appreciated. One staff member commented:

I knew that the chapel didn’t cut it for them. We didn’t feel we had physical plant capability. Well once we…took some of the beds out of service of the rooms that were really not so good…We created this little room and it gets used and people love it. White staff member (Facility 2)

Despite describing more satisfaction with activities, some residents in low-disparity facilities still said more activities were needed, especially on weekends.

###### Mixed-results facility

An issue raised by residents and staff in this facility was that residents with limited English proficiency were unable to participate in many group activities without family engagement and help with translation. The main activities described were bingo and exercise classes, attended by more White than BIPOC residents, and activity volunteers were not proficient in languages other than English. In addition, activities were not modified to include residents from varied racial/ethnic and cultural groups.

##### Culturally appropriate meals and food-related issues

###### High-disparity group

In these facilities, many residents described considerable food-related issues that negatively affected their daily lives. Residents’ concerns centered on several issues: lack of culturally relevant foods, food served late, and food mix-ups. In a facility that served many Asian (Hmong, Vietnamese) residents, residents said that the rice was often dry and improperly prepared. In another facility, several residents said that food was served at inconsistent times and often hours late. One resident commented:

It’s terrible. I mean, I fuss still about the food so much. It’s not so much just the food, it’s an hour late, it’s two hours late. And then sometimes, you got residents eating lunch at 2:30 so at 6 o’clock, no one’s gonna be up to eat because they’re all going to sleep. African American resident (Facility 6)

###### Low-disparity group

Although residents in these facilities had concerns about food, they often said that facility administrators tried to correct food-related issues. In one facility, pork had been served to residents who did not eat pork for cultural/religious reasons. The administrator and Director of Nursing, who was an African American woman, worked with residents and kitchen staff to prevent similar errors. Still, respondents in low-disparity facilities also frequently said that staff could do more to provide culturally sensitive foods. One staff member said:

maybe they could try to accommodate the cultural foods around here. I might not like this, I might not like that, but you know, maybe they could give you a little bit of diversity. The only time that they had anything that was pertaining to us as Black people, like two days in February. African American staff (Facility 4)

###### Mixed-results group

Residents in this group had mixed experiences related to food, with some problems, such as residents being served food they were allergic to or not receiving a meal they requested. Yet, other respondents pointed to efforts administrators made to improve food offerings, such as for residents from other countries.

#### Community level of influence: engagement with BIPOC volunteers and community organizations

##### High-disparity group

We observed limited or no involvement of volunteers or community organizations in this group, and family involvement was also often lacking. The only community activity was church services on Sundays, with primarily White staff. As volunteers in these facilities, we observed a sharp difference in how our team members were treated, with staff frequently questioning our presence. Facilities in this group had stringent volunteer policies for background checks, use of personal phones, and interactions with residents. One staff member shared,

…any prospective volunteers, regardless of where they are located, we have to drive to [location] to get [finger] printed. I will say that has been probably the largest detriment I’ve had to finding volunteers who are willing to actually do it once they realize how the process works. White staff member (Facility 6)

Similarly, when asked about volunteers, an African American staff from Facility 1 said:

I don’t know. I haven’t seen anybody from the community here, so I couldn’t answer that. Which I probably might say, I seriously doubt that the community interacts with them.

##### Low-disparity group

These facilities had a strong volunteer presence and infrastructure to support ongoing community engagement (eg, a volunteer coordinator, annual volunteer appreciation event). One facility had a particularly strong volunteer program where leadership focused on strengthening the diversity of the volunteer pool by partnering with a state agency who participated in a program that offered financial incentives to volunteers from the surrounding community. A staff member commented:

One area where I’ve seen a huge change is in our volunteers. I would say at least 50% of our volunteer force now is African American, where none of them were before. So, people from our [surrounding] community are now volunteering here instead of people from the [protestant denomination] churches who [established the facility]. White staff member (Facility 4)

In this same facility, leadership established a program whereby students from nearby colleges could live on campus (which included senior housing and assisted living services) and volunteer with residents in exchange for reduced rent. One resident commented:

they [students] came from the church over there. A lot of them come. They come with a group from the University too and they talk with us. They know us when they come, and they feel that they have excellent friends with the elderly, and those that are young people in college, they love to come, and we love having them… African American resident (Facility 4)

##### Mixed-results group

Interviews and observations describe limited community involvement in this facility. Staff describe seeing a few church groups who volunteer on Sundays and observations describe 1–2 semiregular volunteers in the facility. When asked, staff also talked about limited volunteer pools:

I’ve seen some, But not a lot. African American staff member (Facility 3)

Many staff recognized the need and challenge of finding more volunteers:

It’d be great if we had more community volunteers. Because a lot of our residents don’t have family members that come and visit. But then I think it’s left up to the staff members to do that, which doesn’t always happen. White staff member (Facility 3)

## Discussion

In this concurrent mixed-methods study, we addressed 2 aims. First, we explored processes contributing to racial/ethnic disparities in QoL in high-proportion BIPOC facilities, and second, we examined how individual, organizational, and community factors affect NH residents’ QoL and the role that between-facility variation plays in these disparities. We were guided by the NIMHD Health Disparities Model (14) and Zubritsky et al.’s framework for LTC QoL (15). Our integration of quantitative and qualitative methods enabled us to document that BIPOC residents report lower QoL than White residents in high-proportion BIPOC NHs, yet that there is variability between these facilities in supporting residents’ QoL. By identifying how different levels of influence impact racial/ethnic disparities in QoL, our results contribute new and actionable knowledge related to improving residents’ QoL in NHs, with policy and practice recommendations.

To the best of our knowledge, this is the first study to identify variability in residents’ QoL between high-proportion BIPOC facilities. High-proportion BIPOC facilities were more likely than other facilities to be for-profit (vs not-for-profit or government owned), have higher proportions of Medicaid share per resident days, and be larger and have lower staffing, consistent with existing literature (13). However, our results also demonstrate differences between the 3 qualitatively identified groups. Specifically, facilities in the “high disparity” group had different organizational characteristics than facilities in the “low disparity” and “mixed results” groups. “High disparity” facilities were all for-profit (vs “low disparity” and “mixed results” groups that were all not-for-profit), had a higher proportion of Medicaid resident days, had fewer private beds, and had lower staffing for direct care staff. The “high disparity” facilities also had a considerably higher proportion of men and a younger resident population. These findings reflect the interplay between facility-level characteristics, organizational culture, and individual QoL, as reflected by the Zubritsky model of QoL (15).

Residents in these 3 types of facilities also had measurably different experiences with QoL with disparities between White and BIPOC residents pronounced by group. These findings demonstrate the need to focus interventions in “high disparity” BIPOC facilities on equity improvements for social activities, food choices, and caregiving from staff, which are vital to residents’ QoL and in which we see the greatest gaps (23).

Our qualitative findings elucidate the quantitative findings, with observational data showing that “high disparity” facilities lacked a culture that valued diversity and engagement, with few family members visiting and none or few volunteers. In “high disparity” facilities, we also found distrust between residents and staff (especially administrators), altercations between residents, and other safety issues such as illegal substance use among residents. Residents in “high disparity” facilities discussed lack of culturally sensitive foods, lack of dignity and respect in their treatment, and receiving disparate care compared with White residents. They also described few activities, limited community outings, and frequent use of racial slurs by other residents. BIPOC staff in the “high disparity” group also discussed frequent use of racial slurs toward them, stressful work environment, with many noting BIPOC residents received worse care. In comparison, facilities in the “low disparity” group had organizational cultures that supported more diversity, including having BIPOC staff in leadership positions and meaningful use of translators. Observations similarly revealed strong volunteer involvement in “low-disparity” facilities, including many volunteers from BIPOC communities. These findings are important in light of the NIMHD framework, which calls attention to the influence of organizational and community-level domains on outcomes.

### Implications of the Findings for Future of NH Care

This mixed-methods study places racial disparities in residents’ QoL and the role of facility racial composition into an important context. Policy and organizational strategies are needed to improve residents’ QoL, particularly for BIPOC residents in high-proportion BIPOC facilities with characteristics (such as for-profit status or more Medicaid-payment residents) that place residents at higher risk for diminished QoL.

First, information on residents’ QoL by race/ethnicity could be systematically collected for all Medicare and Medicaid-certified NHs. Public reporting mechanisms, such as Care Compare (24), could provide an avenue for policy change by reporting QoL by race/ethnicity on state-level report cards to incentivize action among states to address disparities and help BIPOC individuals decide where to receive care. This is reflected in a societal level of influence as per NIMHD framework.

Second, an organizational level of influence is the need to recruit more BIPOC staff for activities and leadership roles (eg, Director of Nursing) to ensure the needs and interests of BIPOC residents are met. Building on observations and interviews, having BIPOC staff in leadership helps elevate the needs, care and concerns of BIPOC residents. BIPOC residents described feeling more comfortable and having more trust to raise care issues with BIPOC leadership and staff, pointing to instances where White residents were treated better or given more leeway by White leadership. Activities staffing levels are associated with increased resident QoL (21,25) and employing BIPOC staff who offer culturally sensitive programing can be an important indicator of facility investment in resident QoL. For example, the “low-disparity” group had the most activity staff and volunteers. Some facilities said they were looking into starting a few cultural activities and including learning days about different cultures. Having more BIPOC activity staff could support many other types of activities that are of cultural significance to residents, and could include culturally significant food in these celebrations.

Third, another key organizational level of influence is that administrators need to expand their facilities’ programming to include a broader range of activities for all residents and activities specifically for BIPOC residents such as music, outdoor outings, and culturally sensitive activities (eg, gardening for Hmong residents).

Fourth, a community and organizational levels are influence reflect the need for facilities to engage residents with diverse racial/ethnic backgrounds in planning food and activities (eg, through Resident Councils) (24), with engagement of BIPOC community volunteers and family members.

Fifth, an individual and organizational levels of influence include staff training to help facilitate relationships for new residents, with particular attention to BIPOC residents without families, residents with limited English proficiency who may be more socially isolated, and those with cognitive impairment (26). Many staff interviewed discussed the benefits of cultural competency training and the need for more opportunities for such training. Examples of training might include culturally competent activity planning, meal planning and in specific skills such as caring for different hair types. Some facilities offered beautician services but no staff knew how to care for different hair textures, and residents had to help each other or rely on family for help. Staff training and organizational policies could also support family and volunteer involvement in the facility (27,28). For example, facilities with a high proportion of BIPOC residents need to leverage BIPOC community-based organizations to combat disparities and promote transparency. To achieve this, facilities need to create a welcoming environment for BIPOC residents and develop incentives for volunteers. Initiatives could involve intergenerational connections with BIPOC younger adults where BIPOC older adults could be mentors.

This study has important limitations. First, the research took place in Minnesota with a NH resident population that is predominately White. Second, the characteristics of NHs that participated in the study’s qualitative component differed from Minnesota facilities in general, with all 6 facilities located in urban areas and having a higher proportion of BIPOC residents than other NHs in the state. These analyses are exploratory in nature. However, no study has examined variability *between* high-proportion BIPOC facilities. Third, our quantitative analyses provide context and comparison for the qualitative findings, and although we present *t*-tests for QoL comparison in the qualitative sample, these comparisons are meant to be descriptive due to lack of statistical power. Fourth, we are also not aiming to disentangle system-level factors such as for-profit ownership or high-proportion Medicaid-payment in creating dis/incentives for cost efficiency and care standards, irrespective of race/ethnicity. Finally, although we utilize the BIPOC term to identify all historically marginalized populations, we recognize that racial/ethnic groups within the BIPOC definition are not homogenous. In quantitative analysis, we examined QoL scores for Black residents in sensitivity analyses (findings were similar). Yet, this study is an important step in understanding mechanisms that impact QoL for BIPOC residents in NHs and recommend that future studies examine QoL for specific racial/ethnic groups, including variability between high-proportion BIPOC NHs. Despite these limitations, this study addresses an important gap in identifying processes that impact racial/ethnic disparities in QoL variability between high-proportion BIPOC facilities, and organizational and community levels of influence that can address these disparities.

In conclusion, this mixed-methods study found that there are important racial/ethnic disparities in residents’ QoL with variability between high-proportion BIPOC facilities. Our results highlight the need for system-level interventions to promote residents’ QoL in NHs, including incentives to engage with community BIPOC organizations and volunteers, and providing more resources to high-proportion BIPOC facilities to support staff training, additional diversified staffing, more culturally sensitive dietary choices, and culturally specific programming, among other options. As NHs increasingly serve a more diverse mix of residents, including growing proportions of BIPOC adults, ensuring equity in QoL for BIPOC residents is an urgent priority if NHs will remain relevant in the future.

## Supplementary Material

igac037_suppl_Supplementary_Material
