# Supplementary material for: Evidence to Inform Policy and Practice: Mechanisms to Address Racial/Ethnic Disparities in Nursing Home Quality of Life
Source: Innov Aging. 2022 May 23;6(4):igac037. doi: 10.1093/geroni/igac037 (PMC9273400; doi:10.1093/geroni/igac037)
Supplement: igac037_suppl_Supplementary_Material [file igac037_suppl_supplementary_material.docx]

**Supplementary Table 1: Description of Quality of Life Domains**

| **Domain** | **Description** |
| --- | --- |
| Summary | Mean of 9 domains below, ignoring domains for which all questions are missing |
| Meaningful Activities | Recreational activities that are enjoyable and meaningful to the resident |
| Food Enjoyment | Is the food enjoyable and varied |
| Environment | Resident’s room and belongings are accessible and safe, offers privacy, access to outdoors |
| Dignity | Are residents treated with respect, e.g. politeness, respect modesty |
| Autonomy | Autonomy over own schedule and care routine |
| Relationships | Quality of relationships with staff and residents |
| Caregiving | Satisfaction with care (e.g., timeliness, consistent caregivers, overall satisfaction) |
| Lack of Negative Mood | Lack of fear, boredom, anger, worry, loneliness |
| Positive Mood | Being relaxed, interested in things, happy |

**Supplementary Table 2: Facility Average Quality of Life by Race/Ethnicity**

|  | **Black** | | **All Other Race/Ethnicity Groups** | | **White** | |
| --- | --- | --- | --- | --- | --- | --- |
|  | **Ref. Group** | **Sample Facilities** | **Ref. Group** | **Sample Facilities** | **Ref. Group** | **Sample Facilities** |
| Summary | 73.10 | 73.57 | 72.01 | 64.70 | 77.45 | 72.28 |
| Food | 62.79 | 50.73 | 67.59 | 55.69 | 73.60 | 67.04 |
| Environment | 82.49 | 88.99 | 82.25 | 76.84 | 87.13 | 83.25 |
| Dignity | 87.98 | 91.21 | 84.11 | 77.81 | 91.39 | 85.67 |
| Autonomy | 75.52 | 82.60 | 73.93 | 70.19 | 80.43 | 74.42 |
| Relationships | 72.14 | 69.73 | 68.43 | 58.30 | 71.38 | 66.71 |
| Caregiving | 68.34 | 69.25 | 67.41 | 62.50 | 73.96 | 68.53 |
| Activities | 72.16 | 71.24 | 69.61 | 58.77 | 74.99 | 69.09 |
| Lack of Neg. Mood | 60.08 | 64.13 | 59.39 | 51.97 | 62.68 | 58.60 |
| Positive Mood | 69.90 | 66.27 | 69.35 | 68.46 | 74.75 | 73.44 |

**RESIDENT INTERVIEW QUESTIONNAIRE**

Hello, my name is XXX. I am a research assistant for the study conducted by the University of Minnesota on racial differences in nursing home care. I will be leading this interview and want to outline a few main points for you.

First, thank you so much for your time. We appreciate your participation and value your input. All of your comments will be strictly confidential and will only be used for research. Your participation will not influence your care at NAME OF FACILITY and we will not share any information with the administration. We ask that you read this form [distribute consent form] and ask any questions you may have before agreeing to be in the study. I will explain a few points on this form after you have read it to make sure you understand what we are asking of you.

The purpose of this study is: to understand minority nursing home residents’ experiences of care and day to day life in the nursing home. Please remember, there are no right or wrong answers. The interview will last about 45 minutes (but we are happy to talk longer!). The interview will be recorded by audiotape, and such recordings will be secured. **Your name will not be used**. We will summarize all the information and your insights without using any names of participants or nursing homes.

Participation in this study is completely voluntary. If you decide to participate, you are free to not answer any question or withdraw from the study at any time. I will stop the interview if a question causes you discomfort and you no longer wish to continue. If you decide to not participate in the study, your care will not be affected in any way. Your feedback will help lead us to a better understanding of the quality of life for minority residents in nursing homes.

After reading the consent form, do you agree to participate in the study. **IF RESPONDENT SAYS YES, GO TO THE LINE BELOW:**

Thank you, I will now turn on the recorder. Please **state** that you agree to participate in the interview.

**I’d like to learn a little bit about how you came to be at [name of facility].**

1. How long have you been here in [name of home]?
2. What was the main reason you moved into [name of facility]?
3. Did you have any choice in selecting [name of home]?

Probes:

- 1. If YES, what are key factors that influenced your choice?
     1. *Did the finances/cost play any role in the selection of a facility?*
     2. *How important was it for you to have religious care in the facility you choose?*
     3. *How important was it for you to be in a facility that has other people of color?*
  2. If NO, *who made the choice for you and how did you end up in this specific facility?*

1. How important was it for you to be in this neighborhood? (if NO to #3, ask whether they like this neighborhood.
   1. Probes*: is this the same neighborhood as where you lived before moving to the facility?*
   2. *If yes, did you consider going elsewhere?*
   3. *If no, does the facility neighborhood matter for the choice of nursing home?*

**I’d like to learn about how you spend your time here.**

1. Tell me about the activities you do in the [name of facility], if any?
   1. *Probes: Do you have something enjoyable to look forward to every day?*
   2. *Are there things to do on the weekends that you enjoy?*
   3. *Do you like the provided activities here?*
   4. Can you enjoy the outdoors when you want to?
   5. Can you find a place to be alone when you want to be alone?
2. How important is it for you to be involved in the facility activities and events?
   1. *Probes: Are you given opportunities to do things that are meaningful for you?*
   2. *What activities would you like to see at [name of facility]?*
3. Can you get the food you like here? Do you look forward to mealtimes here?
   1. *Probes: do you have any input into the food that is served?*
   2. *Do you like the food that is served?*

**I would like to learn about any relationships/friendships you have in this home.**

1. Could you talk about your friends at [name of home], if any?
   - *Probes: Are you friends with any of the other people who live here?*
   - *Are you friends with any staff?*
2. Did you know anybody living at [name of facility] before moving here?
3. How do residents get along with each other? Have you seen residents helping each other? Can you give me examples?
4. What is the role of *race/ethnicity* *or culture* in how residents get along?
5. How much do you feel you can depend on staff in this facility?
   1. *Probes: Do the people who work here know what you like and don’t like?*
   2. *Can you get help when you need it?*
6. Do you feel that staff culture or race/ethnicity influences your relationships with them?
   1. *Probes: Do the staff treat you with respect? Does it differ between white staff/staff of color?*
   2. *Do they listen to what they say? –same as above-*
   3. *Do they treat you with patience? –same as above-*

**I have a couple of questions about your family members.**

1. Do you have any family members visiting you here at [name of facility]? IF so, do they ever participate in any activities?
   1. *Probes: What types of activities?*
   2. *How often do they visit?*
   3. *Do you leave the facility to visit them or does your family pick you up for events, holidays, etc.?*
2. How much do you feel you can depend on your family/friends or people in your community when you have a problem? Why?

**Administration/staff for sense of community**

1. Do you trust the administration (people who run the facility) to make good decisions for you in [name of facility]?
2. Do you feel that residents’ race/ethnicity plays a role in the care they receive from staff? If so, please give examples.

**Tension**

1. Have you had any tension with residents in [name of facility]?
2. Do you feel that having many residents from different racial groups in [name of facility] affects how people get along?

*Do people tend to be friends with those from their own cultural or racial group? Tell us more about it.*

1. Do you anticipate staying in NAME OF FACILITY for an extended period of time?

**Now I’d like to talk about how satisfied you are with care in this facility.**

1. All things being considered, how satisfied are you with your life as a whole these days? Why or why not?
2. How at home do you feel in [name of facility] overall? What about in your room?
   1. *Would you recommend (Name of Facility) to someone who needs care?*

**Other**

1. Are there other topics that should be discussed that were not included here?

YOU CAN FILL OUT THE FACT SHEET AT THE END OF THE INTERVIEW. DO NOT GIVE THIS TO THE PARTICIPANT: FILL IT OUT YOURSELF.

**Fact Sheet**

Name: _______________________________________________

Name/Address of Nursing Home: ___________________________________

Race/Ethnicity: __________________________________________

Gender: ______________

Date of Interview: ______________________ Interviewer(s): ___________________

Time Begun: __________________________________________________________

Time Ended: __________________________________________________________

Other Interview Details: _________________________________________________

**STAFF INTERVIEW QUESTIONNAIRE**

Hello, my name is **XXX**. I am a research assistant for the study conducted by the University of Minnesota on racial differences in nursing home care. I will be leading this interview and want to outline a few main points for you, as well as provide you with the appropriate documentation before beginning.

First, thank you so much for your time. We appreciate your participation and value your input. All of your comments will be strictly confidential and will only be used for research. Your participation will not influence your employment at NAME OF FACILITY and we will not share any information with the administration. We ask that you read this form and ask any questions you may have before agreeing to be in the study [**GIVE THEM THE CONSENT FORM**].

The purpose of this study is: to understand minority nursing home residents’ experiences of care and life in the nursing home. We will be talking to residents but we also believe it is very important to understand the perspectives of those who work in nursing homes-direct care staff, administration, and other staff members. Your thoughts and experiences are **incredibly important** and there are no right or wrong answers.

The interview will last 30-45 minutes. The interview will be recorded by audiotape, and such recordings will be secured. Your name will not be used. We will summarize all the information and your insights but without using any names or respondents or nursing homes.

After reading the consent form, I would like to know if you agree to participate in the study. **IF RESPONDENT SAYS YES, GO TO THE LINE BELOW:**

Thank you, I will now turn on the recorder. Please **verbally state** that you agree to participate in the interview.

**Before we get to the main topics, we would like a bit of information about you.**

1. How did you come to work in NAME OF HOME?

1. Tell me a bit about your work experience. Did you work in places other than NAME OF HOME?
2. How many years or months have you been at NAME OF HOME?

_________ Timeframe in your current position

_________ Timeframe working at this nursing home

1. What is your educational background? (have them say it, but if they don’t’ say it, ask:
   1. High school degree/GED
   2. Associate’s degree (specify)

____________________

- 1. Bachelor’s degree (specify)

____________________

- 1. Master’s degree (specify)

____________________

- 1. Certification (specify)

**We all know that work environment is very important for one’s ability to deliver best care. I’d like your perspectives on the work culture here in NAME OF HOME.**

1. Can you talk about the environment of your work? (Probes: communication between staff, communication between management & staff, communication between departments, staffing levels, etc.)
2. Does the facility have resources in place to provide best care for all residents, particularly residents from different racial/ethnic or cultural backgrounds?
3. How much freedom do staff have (if any), to alter work routines based on resident’s preferences and needs? How do cultural differences influence residents’ preferences and needs, if at all (please give examples)?

**Probes:**

Ask staff if they feel managers/supervisors are aware of needing extra time and how they react when staff feels they need extra time to meet cultural needs when there are other demands

1. Do you feel that the care provided to residents in NAME OF HOME aims to be sensitive, respectful, and inclusive to the various cultures of the residents? Why or why not?
2. What would you say is one most challenging and most rewarding aspect of your job?

**We know that for many nursing home residents, staff members become their second family. We want to learn more about staff relationships with residents in NAME OF FACILITY.**

1. Have you observed residents and staff forming meaningful relationships? Can you give examples?
2. Tell me about your own relationship with residents? Are there residents with whom you have close rapport? Could you describe a case when it was challenging to build a relationship and reasons for it?
3. From your perspective, do you feel that residents receive good quality of life/quality of care in this facility? Why or why not?

**This last set of questions is about care for minority residents. As you know, our study tries to understand reasons why minority nursing home residents report lower quality of life than white residents. We’d like your perspectives and experiences when interacting with minority residents.**

1. Do you feel that resident ethnicity, race, or cultural background impacts staff ability to deliver care? Could you give examples?

**Probes:** have you heard minority residents complain about their life in the facility. If so, what are these complains typically about? Food? Relationships with staff? Other?

Do you feel that resident’s ethnicity, race, or cultural background influences the quality of the interactions between staff and residents? Please give examples.

1. Do you feel that minority residents are offered the opportunity to be involved in meaningful (culturally-relevant) activities? Can you discuss? Are these formal activities or mostly informal (e.g., individual staff interactions)?
2. What are your recommendations for best standards in providing care for individuals from diverse racial/ethnic and cultural groups? Are there specific resources that facilities need to have? Any specific staffing requirements?

**Finally, our work shows that residents’ satisfaction with care is also influenced by the neighborhood where the nursing home is located. We want to hear your thoughts about the role of neighborhoods for resident satisfaction of care.**

1. What is your general experience with the neighborhood/community in which NAME OF HOME is located? Are there any particular challenges or issues in this neighborhood that could impact residents’ quality of life?
   1. What are the opportunities or barriers for residents to interact with community members? [Probes: availability of volunteers, involvement from local community organizations, environment in the neighborhood]

**Other**

1. Are there other topics that should be discussed that were not included here?

YOU CAN FILL OUT THE FACT SHEET AT THE END OF THE INTERVIEW. DO NOT GIVE THIS TO THE PARTICIPANT: FILL IT OUT YOURSELF.

Fact Sheet

Name: _______________________________________________

Name/Address of Nursing Home: ___________________________________

Job Title in Nursing Home: __________________________________________

Gender: ______________

Date of Interview: ______________________ Interviewer(s): ___________________

Time Begun: __________________________________________________________

Time Ended: __________________________________________________________

Other Interview Details: _________________________________________________
